# Enhancing the Signature Rose Aroma of *Kluyveromyces marxianus*-Fermented Milk Beer via Adaptive Laboratory Evolution

**DOI:** 10.3390/foods15020229

**Published:** 2026-01-08

**Authors:** Chen Xing, Youming Tan, Xinchi Jiang, Wenlu Li, Qihao Wang, Zihao Liu, Hong Zeng, Yanbo Wang

**Affiliations:** 1 School of Food and Health, Beijing Technology and Business University, Beijing 100048, China; 2330202076@st.btbu.edu.cn (C.X.); 2431031001@st.btbu.edu.cn (Y.T.); 18515391598@163.com (X.J.); liwenlu13@163.com (W.L.); 2531031037@st.btbu.edu.cn (Q.W.); 2330201009@st.btbu.edu.cn (Z.L.); 2 Key Laboratory of Geriatric Nutrition and Health, Beijing Technology and Business University, Ministry of Education, Beijing 100048, China

**Keywords:** milk beer, adaptive laboratory evolution, comparative genomics analysis, flavoromics, quantitative descriptive analysis, *Kluyveromyces marxianus*

## Abstract

Milk beer, a modern Chinese dairy beverage, is usually fermented by the co-culture of lactic acid bacteria (LAB) and *Kluyveromyces marxianus* (*K. marxianus*), with the latter known for its ability to produce aroma compounds. However, the accumulation of lactic acid produced by LAB can inhibit the growth of *K. marxianus*, which inevitably hinders the diversity and intensity of flavor compounds in milk beer. In this study, adaptive laboratory evolution (ALE) was applied to the parental strain *Kluyveromyces marxianus* CICC1953 (Km-P) under different concentrations of lactic acid to obtain an evolved strain Km-ALE-X20 with enhanced acid tolerance and increased titer of phenylethyl alcohol, which has a floral, rose-like aroma. Km-ALE-X20 demonstrated a 16-fold increase in OD_600_ and a 28-fold increase in phenylethyl alcohol production compared with Km-P in chemically defined medium (CDM) containing 20 g/L lactic acid. Comparative genomics analysis suggested that mutated genes *CTA1*, *TSL1*, *ERG2* were related to enhanced acid tolerance, while *ARO8*, *ARO9*, *FKS2* were related to increased production of aroma compounds. Furthermore, Km-ALE-X20-fermented milk beer showed 33.87% and 32.43% higher production in alcohol and ester compounds than that of Km-P-fermented milk beer. Interestingly, sensory analysis showed that while Km-ALE-X20-fermented milk beer had higher sensory scores for rose and fruity aroma attributes, Km-P-fermented milk beer possessed a more balanced aroma profile. This paper highlights the first application of ALE to enhance the signature rose aroma of *K. marxianus*-fermented milk beer and provides an efficient framework for ALE-based breeding of aroma-producing food microorganisms.

## 1. Introduction

Milk beer, a modern and popular Chinese dairy beverage, is commonly produced from whole and skim milk powder or whey through fermentation of a co-culture of lactic acid bacteria (LAB) and yeast [1]. This nutritionally rich beverage is characterized by fresh, creamy, fruity, and malty notes that offer a well-balanced sweet and sour taste, complemented by a delicate and abundant foam. As a result, milk beer has gained popularity among young consumers [2]. Recently, companies like Xinjiang Tianrun have begun utilizing the unconventional yeast, *K. marxianus* for milk beer production [3]. This yeast is capable of metabolizing lactose and is already well-established in the fermentation of dairy products like kefir, yogurt, and cheese [4]. It is also a valuable industrial microorganism due to the ability to produce aromatic compounds such as phenylethyl alcohol and phenylethyl acetate [5]. Given the long history of safe consumption of dairy products, *K. marxianus* is generally regarded as a safe (GRAS) strain [6]. It can improve the quality of fermented products by reducing ethanol levels, enhancing aroma complexity, and decreasing acidity [7,8].

Co-culture fermentation involving LAB and yeasts is a widely employed strategy to enhance the flavor quality of food products [9]. However, the lactic acid produced by LAB is a weak organic acid whose dissociation depends on the environmental pH and its pKa. Under acidic conditions (where the environmental pH is lower than the pKa of lactic acid, approximately 3.86), the acid predominantly exists in its undissociated form, which can freely enter the cytoplasm through passive diffusion across the yeast cell membrane. Because the intracellular pH is close to neutral and higher than the pKa of lactic acid, the lactic acid molecules dissociate into protons (H^+^) and lactate ions. The accumulation of protons disrupts intracellular pH homeostasis, alters the structure and activity of key enzymes [10], which in turn inhibits the normal physiological metabolism and fermentation activity of *K. marxianus*, thereby indirectly reducing the diversity and content of aroma compounds in milk beer. Therefore, enhancing the acid tolerance of *K. marxianus* is essential for maintaining its fermentation activity and aroma production ability. Several strategies have been explored to improve acid tolerance in yeast strains, including random mutagenesis, metabolic engineering and environmental adaptation. Random mutagenesis can generate a large number of acid tolerant mutants; however, it often suffers from limitations such as unpredictable mutation directions and phenotypic instability [11]. Although metabolic engineering enables targeted modification of specific pathways, it requires detailed knowledge of acid stress mechanisms. Moreover, genetically modified microorganisms are rarely commercialized in traditional food and probiotic industries [12]. Therefore, there is a clear need for an alternative strain improvement strategy that can enhance acid tolerance while maintaining phenotypic stability and regulatory compatibility for food applications.

Adaptive laboratory evolution (ALE) has been widely applied in microbial strain improvement under specific environmental stressors [13]. This process involves the long-term cultivation of microorganisms under controlled stress conditions, inducing genetic mutations that lead to desired phenotypic adaptations relevant to the environmental challenges faced [14]. A key advantage of ALE is that it does not require prior genetic knowledge, while providing basic information on molecular aspects of evolution and genotype–phenotype interconnections [15]. ALE is generally considered a safe and effective transformation method. The evolved strains can be directly applied to the production of fermented foods [16]. Recently, many studies have utilized ALE to domesticate food-related microbial strains. For example, one study combined ALE with atmospheric and room temperature plasma to obtain a *Saccharomyces cerevisiae* strain with enhanced lactic acid tolerance and improved aroma production, The application of this strain improved the flavor quality of baijiu [17]. Another study employed ALE to enhance the acid tolerance of *Fructilactobacillus sanfranciscensis*, resulting in increased production of key metabolites crucial for bread flavor [14]. Similarly, an ALE-modified strain of *Pichia pastoris*, which was cultivated in 100 g/L NaCl, resulted in a 0.64-fold increase in phenylethyl alcohol production compared to that produced by the wild-type [18].

Improving the flavor quality of milk beer is of considerable practical significance for the dairy fermentation industry. With the rapid expansion of the market for novel and high-quality fermented dairy products, enhancing the distinctive rose-like aroma of milk beer increases its sensory appeal, improves product differentiation, and adds commercial value. Meanwhile, ALE represents a non-genetically modified, efficient, and industrially applicable strategy for tailoring flavor traits in food-related microorganisms. This study employed adaptive laboratory evolution and obtained an evolved *K. marxianus* strain with improved lactic acid tolerance and enhanced aroma production ability. To determine the genetic basis of the observed phenotypic changes, comparative genomic analysis was performed between the parental and evolved strains. The evolved strain was further applied to the milk beer system, and the volatile organic compounds in the milk beer were analyzed using flavoromics approaches. Furthermore, the variable importance in projection (VIP) scores based on the partial least squares-discriminant analysis (PLS-DA) model was utilized to identify the key flavor compounds. Finally, quantitative descriptive analysis (QDA) was employed to quantify the sensory attributes of the milk beer, revealing differences in aroma profiles between the parental and evolved strains-fermented milk beer based on the flavor wheel. Overall, this study presents and validates an efficient strategy to reinforce the signature rose aroma and flavor diversity of fermented dairy products through integrative ALE, flavoromics and sensory evaluation approaches.

## 2. Materials and Methods

### 2.1. Strains and Cultivation Conditions

The parental strain *Kluyveromyces marxianus* CICC1953 (Km-P) was obtained from the China Center of Industrial Culture Collection (CICC, Beijing, China). It was stored at −80 °C in 40% (*v*/*v*) glycerol in the laboratory of the Food Flavor and Health Innovation Team at Beijing Technology and Business University. The preserved yeast strain was activated on solid Yeast Extract Peptone Dextrose (YPD) medium (10 g/L yeast extract, 20 g/L peptone, 20 g/L glucose, and 20 g/L agar) and incubated at 30 °C for 48 h. A single colony was inoculated into YPD medium and cultured at 30 °C with shaking at 200 rpm until the mid-logarithmic growth phase was reached. Subsequently, 2% (*v*/*v*) of the culture was inoculated into a modified fermentation medium (20 g/L glucose, 5 g/L yeast extract, 0.4 g/L MgSO_4_·7H_2_O, 2 g/L KH_2_PO_4_, and 5 g/L phenylalanine), as described by previous study [19], for phenylethyl alcohol production.

### 2.2. Adaptive Laboratory Evolution

To determine the optimal stress conditions for ALE, a preliminary experiment was conducted to evaluate lactic acid tolerance of Km-P. Specifically, the strain was cultivated in shake flasks containing lactic acid at concentrations of 0, 5, 10, 15, and 20 g/L. Both growth (OD_600_) and phenylethyl alcohol production were assessed. The concentration of phenylethyl alcohol was determined using a solvent extraction method, with detailed procedures and the standard curve provided in Table S1. The ALE experiment was carried out using an automatic microbial adaptive evolution instrument (EVOL cell, Luoyang Huaqing Tianmu Biotechnology Co., Ltd., Luoyang, China). Prior to ALE, the strain was cultured to reach the mid-to-late logarithmic growth phase in modified fermentation medium and inoculated into the reactor at a 2% (*v*/*v*) inoculum. The reactor was connected via tubing to two media reservoirs: one containing 200 mL of fermentation medium without lactic acid, and the other containing 200 mL with 20 g/L lactic acid. The experimental parameters were set as follows: culture temperature at 30 °C, oxygen concentration at 10%, detection wavelength at 600 nm, inoculation volume at 2% (*v*/*v*), lactic acid concentration gradient from 0 to 20 g/L, passage interval of 20 h, and detection interval of 2 h. After setting the parameters, the ALE experiment was initiated. When the evolving culture exhibited stable growth at the final lactic acid concentration (20 g/L), it was collected using a sterile syringe and stored at −80 °C. The evolved strain was designated as Km-ALE-X20.

### 2.3. Phenotypic Validation

Km-P and Km-ALE-X20 were selected to evaluate growth performance and aroma production ability. After activation, each strain was inoculated into fermentation media without lactic acid and with 20 g/L lactic acid, respectively. The cultures were incubated at 30 °C with shaking at 200 rpm until they reached the stationary phase. The fermentation broth was taken every 2 h to measure OD_600_ values for monitoring cell growth. Additionally, fermentation broth was taken at 18, 24, 30, and 36 h for the determination of phenylethyl alcohol production, following the procedure described in Section 2.2.

### 2.4. Comparative Genomics Analysis

The genome of *K. marxianus* DMKU3-1042 (https://www.ncbi.nlm.nih.gov/datasets/genome/GCF_001417885.1/, accessed on 10 September 2024) was used as the reference genome [20]. Whole-genome resequencing of cell pellets from both Km-P and Km-ALE-X20 strains was performed by Shanghai Majorbio Bio-Pharm Technology Co., Ltd. (Shanghai, China). The DNA of the two strains was extracted using the CTAB method, and its quality was assessed by agarose gel electrophoresis and spectrophotometry. High-quality DNA was fragmented by ultrasonication into random fragments. End-repair, 3′ A-tailing, and adaptor ligation were then performed. DNA fragments of approximately 350 bp were selected using magnetic beads and subjected to PCR amplification to construct sequencing libraries. After quality control, the libraries were sequenced on an Illumina NovaSeq X Plus™ (Illumina, San Diego, CA, USA) platform using the PE150 paired-end sequencing strategy, generating paired-end reads with a total length of 300 bp. Raw reads were subjected to quality filtering to remove low-quality sequences, resulting in high-quality clean reads. These clean reads were then aligned to the reference genome using BWA-MEM. The resulting BAM files were processed according to the GATK Best Practices workflow, including duplicate marking, base quality score recalibration (BQSR), and indel realignment. Single nucleotide polymorphisms (SNPs) and insertions/deletions (indels) were identified using GATK HaplotypeCaller to characterize the genetic variations. Annotation of all variants was performed with SnpEff. Genes with mutations containing the obtained SNPs and indels were extracted. Subsequently, Gene Ontology (GO) and Kyoto Encyclopedia of Genes and Genomes (KEGG) enrichment analyses were conducted using the Metascape online platform (https://www.metascape.org/, accessed on 28 September 2024).

### 2.5. Milk Beer Preparation

The production of milk beer was performed in three stages, including preparation of fermented milk, preparation of primary acid emulsion, and final fermentation. Reconstituted milk was prepared by dissolving 11.5% (*w*/*w*) whole milk powder (Fonterra Co-operative Group Limited, Beijing, China) and 6.5% (*w*/*w*) sucrose (Guangzhou Fuzheng Donghai Food Co., Ltd., Beijing, China) in sterile distilled water at 60 °C. The mixture was homogenized using a high-pressure homogenizer (SRH, Shenlu, Shanghai, China) at 20 MPa, the homogenized mixture was then pasteurized at 95 °C for 5 min and cooled to 37 °C [21]. Subsequently, 0.0012 g of commercial starter culture Chr. Hansen YoFlex Premium 5.0 (Horsholm, Denmark) [22], containing *Streptococcus thermophilus* and *Lactobacillus delbrueckii* subsp. *bulgaricus*, was inoculated into the reconstituted milk and fermented at 37 °C until the fermentation endpoint (pH = 4.6) was reached. To prepare the primary acid emulsion, dry food ingredients, including 0.4% (*w*/*w*) sodium carboxymethyl cellulose (Shandong Zhongyuan Biotechnology Co., Ltd., Shandong, China), 6.5% (*w*/*w*) sucrose, and 0.1% (*w*/*w*) food-grade L-phenylalanine (Zhongyan Ingredients Trading Co., Ltd., Zhengzhou, China) were dissolved in 62% (*w*/*w*) water, and then 31% (*w*/*w*) fermented milk was incorporated and thoroughly mixed. For milk beer fermentation, the *K. marxianus* cells were collected by centrifugation (8000 rpm/min, 15 min) and washed twice with PBS buffer. The cell pellet was then resuspended and inoculated into the primary acid emulsion at 1.5 × 10^6^ CFU/mL. Subsequently, fermentation was carried out in a water bath at 30 °C for 0, 10, 24, 48, and 72 h. 

### 2.6. Flavoromics Analysis

#### 2.6.1. Sample Preparation and Treatment

Firstly, 5 g of milk beer samples were transferred into a 20 mL amber vial equipped with a white silicone septum cap. Secondly, 0.5 g of NaCl was added to each vial. Thirdly, 10 μL of 2-methyl-3-heptanone (Sigma-Aldrich Trading Co., Ltd., Shanghai, China) with a concentration of 0.0816 mg/mL was added as an internal standard. The samples were then placed in a water bath and equilibrated at 45 °C for 30 min. Volatile organic compounds were extracted using a DVB/C-WR/PDMS-coated SPME Arrow fiber (Shimadzu Smart SPME Arrow, 20 mm length × 1.1 mm outer diameter, 120 μm film thickness) at 45 °C for 30 min. Following extraction, the fiber was introduced into the GC injection port and thermally desorbed at 230 °C for 5 min, with the injector temperature maintained at 250 °C.

#### 2.6.2. GC-MS/MS Conditions

The analysis was performed using a GCMS-TQ8050 (Shimadzu Corporation, Kyoto, Japan) NX triple quadrupole gas chromatograph-mass spectrometer equipped with an SH-PolarWax capillary column (60 m × 0.25 mm i.d., 0.25 μm film thickness). Helium was used as the carrier gas at a constant flow rate of 1.0 mL/min. Splitless injection was employed with the injector temperature maintained at 250 °C. Chromatographic separation was achieved using the following temperature program: the oven was held at 40 °C for 5 min, then ramped to 200 °C at 3 °C/min, and finally maintained at 200 °C for an additional 5 min. The mass spectrometer was operated in electron ionization (EI) mode at 70 eV. The ion source and interface temperatures were set at 200 °C and 250 °C, respectively. The mass spectrometer was operated in full-scan mode with a mass range of 35–600 *m*/*z*.

#### 2.6.3. Qualitative and Quantitative Analysis

The volatile organic compounds in milk beer were qualitatively analyzed by mass spectrometry characterization using the NIST20 mass spectral database and retention index (RI). Quantitative analysis of the volatile compounds was performed using the internal standard method. The odor activity value (OAVs) was calculated as the ratio of the concentration of a volatile compound to its odor threshold in water. Typically, an OAV ≥ 1 suggests that a compound contributes to the aroma, with higher OAVs indicating stronger odor intensities and a greater contribution to the overall aroma profile. In this study, the thresholds for all odor-active compounds were obtained from the reference book [23].

### 2.7. Quantitative Descriptive Analysis

A sensory evaluation panel comprising ten trained assessors (5 males and 5 females, aged 23–27) was assembled. Through group discussion, a consensus-based sensory lexicon was developed, including definitions of aroma attributes, reference standards, and corresponding intensity scales. Assessors were trained in pre-experimental sessions using olfactory reference compounds identified from the literature. Sensory evaluation was conducted using quantitative descriptive analysis (QDA). Each assessor received both reference and test samples, with test samples coded using randomized three-digit numbers. The intensity of each aroma attribute was rated relative to the corresponding reference standard. Seven sensory descriptors were evaluated: rose aroma, fruity, green aroma, fermented, creamy, buttery, and milky, with each aroma attribute rated on a continuous scale (0 points indicating the lowest intensity and 9 points indicating the highest intensity). Each analysis was performed in triplicate, and the average values was obtained as the final score of the milk beer samples and visualized in a radar chart. All procedures were developed based on modifications of previously reported methods [24].

### 2.8. Statistical Analysis

All measurements were performed in triplicate for each group. One-way analysis of variance (ANOVA) was conducted using SPSS 26.0 (IBM Corporation, Armonk, NY, USA). Principal component analysis (PCA) and partial least squares discriminant analysis (PLS-DA) of the volatile compounds in milk beer were performed using SIMCA 14.1 and the MetaboAnalyst platform. Bar charts, radar charts, and pie charts were created using Origin 2024. Heatmaps were generated using TBtools (Version 2.056, South China Agricultural University, Guangzhou, China). Two-way ANOVA was performed using Prism (Version 8.0.1, GraphPad Software Inc., San Diego, CA, USA).

## 3. Results and Discussion

### 3.1. Determination of Flavor-Enhanced Strain by ALE

**Figure 1:**
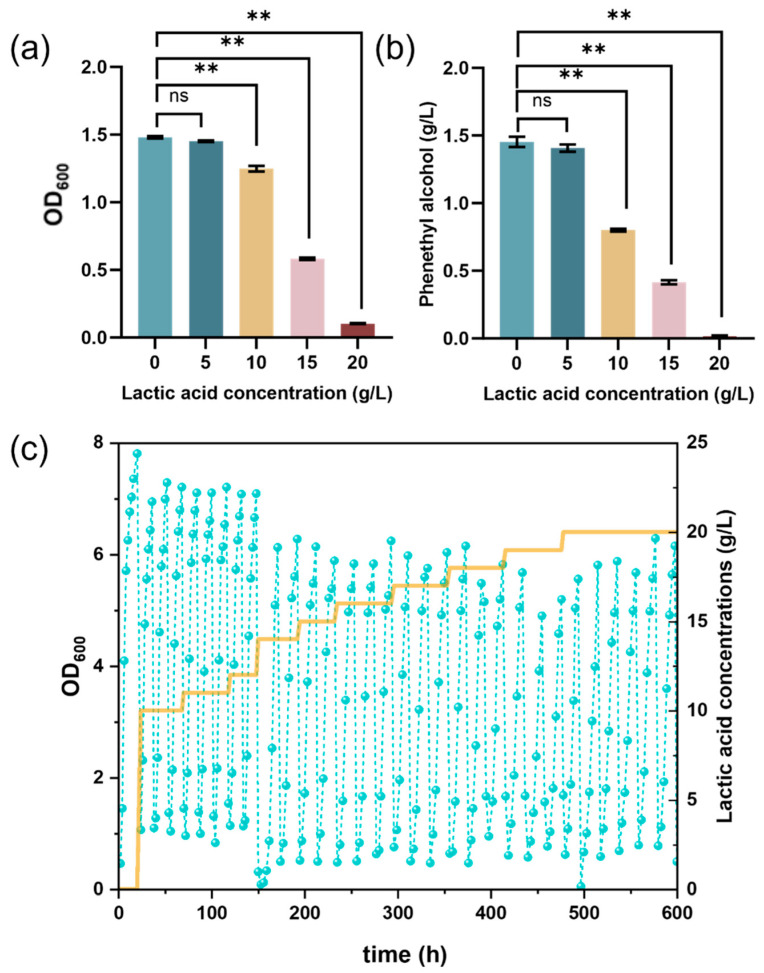
Adaptive laboratory evolution of *K. marxianus* (**a**): Growth of Km-P under different lactic acid concentration. (**b**): Phenylethyl alcohol production of Km-P under different lactic acid concentration. The blue line represents the OD_600_ of the strain, and the yellow line represents the lactic acid concentration in the reaction system. (**c**): ALE of Km-P under lactic acid stress. The error bars represent standard deviation from the mean (*n* = 3 independent cultures). Data were analyzed using one-way ANOVA followed by Welch’s test and Tamhane’s T2 test. ns indicates no significant difference, ** *p* < 0.05, *p* < 0.01.

The lactic acid tolerance and associated phenylethyl alcohol production of the parental strain *K. marxianus*-P (Km-P) were evaluated to determine the appropriate ALE conditions (Figure 1a,b). 5 g/L lactic acid had no significant inhibitory effect on cell growth or phenylethyl alcohol production. However, both parameters were significantly suppressed at 10 g/L (*p* < 0.01), and inhibition became highly significant at concentrations above 15 g/L (*p* < 0.01). Accordingly, shorter cultivation times were used for 0–10 g/L lactic acid and prolonged cultivation times for 10–20 g/L to ensure stable growth throughout the ALE passages (Figure 1c). Km-P was subjected to 600 h of ALE, with the lactic acid concentration increased stepwise from 0 to 20 g/L. The culture exhibited an initial decline in OD_600_ followed by a progressive recovery. During the first 150 h, normal cell growth of Km-P was observed at 0–12 g/L lactic acid concentration. However, when the lactic acid concentration increased to 14–18 g/L, growth inhibition was observed as indicated by a moderate decrease in OD_600_ and fluctuations in growth status. At 19 g/L lactic acid, a marked growth inhibition was observed, with the OD_600_ dropping from 7.81 to 4.90, representing a 37.26% reduction. Nevertheless, after continued enrichment for 180 h, the OD_600_ increased from 4.90 to 6.28. This growth recovery suggested that the culture gradually adapted to the high lactic acid environment and eventually acquired tolerance to lactic acid concentrations up to 20 g/L.

**Figure 2:**
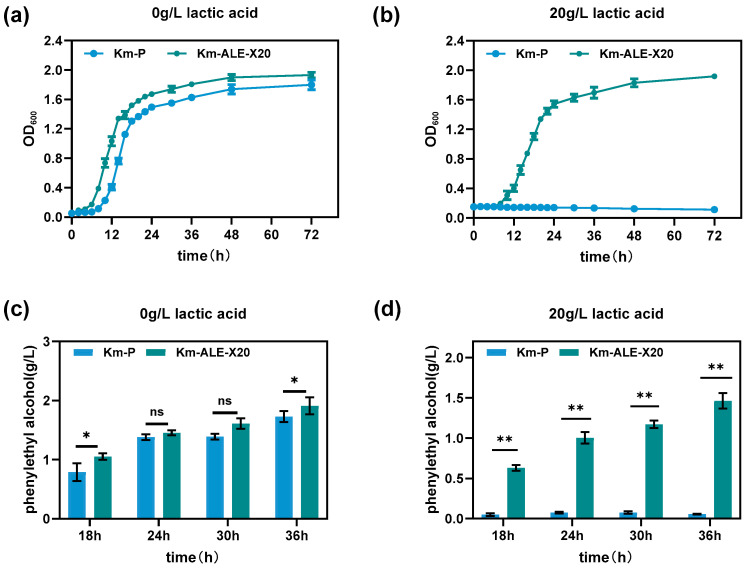
Comparison of cell growth and phenylethyl alcohol production capacity between the parental and evolved strains. (**a**,**b**): Growth of Km-P and Km-ALE-X20 at 0 g/L and 20 g/L lactic acid concentrations. (**c**,**d**): Phenylethyl alcohol production by Km-P and Km-ALE-X20 at 0 g/L and 20 g/L lactic acid concentrations. Experiments were performed in triplicate. The error bars represent standard deviation from the mean (*n* = 3 independent cultures). Statistical significance analysis was conducted using two-way ANOVA followed by Sidak’s multiple comparisons test. ns indicates no significant difference, * *p* < 0.05, ** *p* < 0.01.

To better understand the phenotypic changes resulting from ALE, the cell growth and phenylethyl alcohol production capacity of the evolved strain were assessed. As shown in Figure 2a,b, both Km-P and Km-ALE-X20 exhibited comparable growth trends in lactic acid-free chemically defined medium (CDM), with Km-ALE-X20 displaying slightly better growth compared with Km-P. However, at 20 g/L lactic acid, Km-P exhibited complete growth inhibition, while Km-ALE-X20 maintained robust growth. At 72 h, the OD_600_ of Km-ALE-X20 was approximately 16-fold higher than that of Km-P. The comparison of phenylethyl alcohol production is shown in Figure 2c,d. No statistically significant differences in phenylethyl alcohol production were observed between Km-P and Km-ALE-X20 in lactic acid-free CDM, except at 18 and 36 h (*p* < 0.05). However, under 20 g/L lactic acid stress, Km-ALE-X20 exhibited significantly higher phenylethyl alcohol production than that of Km-P throughout the fermentation process (*p* < 0.01). At 72 h, the phenylethyl alcohol production of Km-ALE-X20 was 28.2-fold higher than that of Km-P. Collectively, the enhanced cell growth and phenylethyl alcohol production capacity of Km-ALE-X20 under 20 g/L lactic acid provide direct evidence that ALE improved the lactic acid tolerance and aroma production capacity of *K. marxianus*.

### 3.2. Identification of Mutated Genes Associated with Enhanced Phenotypes

**Figure 3:**
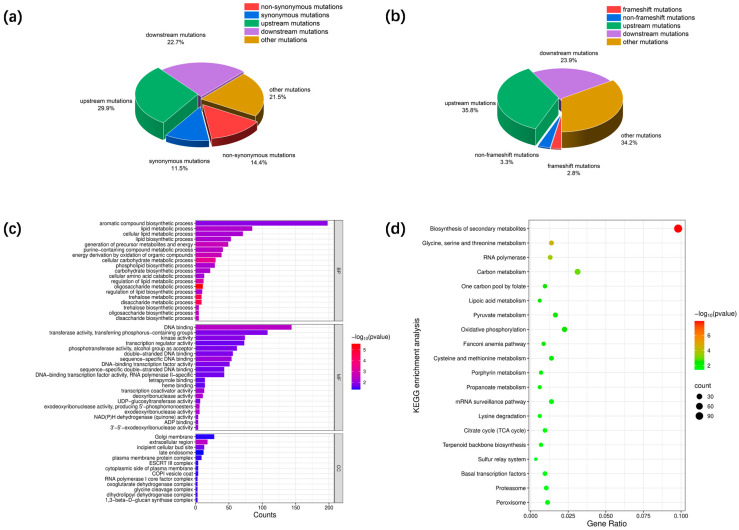
Comparative genomic analysis: variant detection and enrichment analysis. (**a**) Analysis of SNPs. (**b**) Analysis of indels. (**c**) GO enrichment analysis (**d**) KEGG enrichment analysis.

To elucidate the regulatory mechanisms of enhanced lactic acid tolerance and aroma production capacity, comparative genomic analysis was conducted between Km-P and Km-ALE-X20. A total of 1551 protein-coding genes were identified, of which 1210 genes were functionally annotated. During the ALE process, genomic variations in the evolved strain could arise through multiple mechanisms, including the accumulation of point mutations and structural variations resulting from insertions, deletions, horizontal gene transfer, and recombination-dependent intragenomic rearrangements [25]. SNPs represent the predominant form of genetic variation in microbial genomes [26]. A total of 8368 SNPs were identified (Figure 3a), including 1208 (14.4%) nonsynonymous mutations and 965 (11.5%) synonymous mutations. Among the nonsynonymous mutations, 1188 were missense mutations, which can alter protein structure and potentially affect their functions [27]. In addition, 2504 (29.9%) SNPs were located in upstream regulatory regions of genes, while 1899 (22.7%) were located in downstream regulatory regions. A total of 4367 indels were identified (Figure 3b), including 123 (2.8%) frameshift mutations and 144 (3.3%) non-frameshift mutations. Additionally, 1565 (35.8%) indels were located in upstream regulatory regions of genes, and 1045 (23.9%) were located in downstream regulatory regions. The high proportion of SNPs and indels in upstream regions suggested that these mutations might have had a significant impact on transcriptional regulation and gene expression levels, potentially affecting cellular function and phenotypic traits [28]. These results suggest that the enhanced lactic acid tolerance and aroma production capacity of Km-ALE-X20 were achieved through a reprogramming of the transcriptional regulatory network.

Comparative genomic analysis between Km-P and Km-ALE-X20 identified ten candidate genes associated with enhanced acid tolerance and ten candidate genes related to enhanced biosynthesis aroma compounds (Table 1). These mutated genes were further classified into two categories according to whether they caused changes in protein sequences. Protein-altering mutations included missense and frameshift mutations, whereas non–protein-altering mutations comprised synonymous and non-frameshift mutations. Mutated genes associated with acid resistance were categorized into four major functional groups, including antioxidant response and reactive oxygen species (ROS) scavenging, membrane stability maintenance, proton pumps and pH homeostasis, and the trehalose metabolic pathway. Under lactic acid stress, the accumulation of ROS disrupts cellular homeostasis [29]. *CTA1*, *YBP1*, and *POS5* were involved in mitigating oxidative stress. *POS5* encodes a mitochondrial NADH kinase [30]. Km-ALE-X20 likely enhanced the enzymatic activity of *POS5* to provide a more sufficient supply of NADPH for the glutathione and thioredoxin antioxidant system, thereby strengthening the overall cellular reducing capacity. *YBP1* acts as an oxidative stress sensor. Upon exposure to H_2_O_2_, it forms a ternary complex with the transcription factor *Yap1* and the peroxidase *Gpx3* [31]. In Km-ALE-X20, this signaling module may have enhanced the efficiency of *Yap1* oxidation and nuclear translocation, thereby upregulating the expression of antioxidant genes. *CTA1* encodes catalase that directly converts H_2_O_2_ into water and oxygen, thereby limiting the propagation of oxidative damage [32]. Membrane lipids and sterols are essential for maintaining the barrier properties of the plasma membrane under lactic acid or low-pH stress [33]. *IPT1* regulates sphingolipid composition, affecting membrane fluidity, proton permeability, and overall membrane integrity [34]. In Km-ALE-X20, regulation of *IPT1* may have altered sphingolipid composition to mitigate acid-induced damage. *ERG2* and *ERG7* are key components in sterol biosynthesis that influence sterol structure [35]. Km-ALE-X20 likely adjusted the sterol composition to reduce membrane fluidity, thereby limiting the passive diffusion of lactic acid in their undissociated form. Low pH and weak acid stress leads to proton accumulation, disrupt membrane potential, and inhibit cell growth [36]. *VMA16*, along with *VMA3* and *VMA11*, forms a proton channel that utilizes ATP hydrolysis to actively transport H^+^ into vacuoles [37]. Km-ALE-X20 appeared to enhance this system to reduce intracellular acid load. *PDR12* is an acid-responsive ABC transporter whose expression is significantly upregulated under weak acid stress [38]. Km-ALE-X20 likely utilized this transporter to export lactic acid out of the cell, thereby alleviating intracellular acidification. The trehalose metabolic pathway plays a crucial role in stress resistance. *TSL1* is involved in the biosynthesis of trehalose [39]. Under long-term acid stress selection, Km-ALE-X20 enhanced lactic acid tolerance by regulating the balance of trehalose metabolism. *TRE2* participates in trehalose degradation, providing carbon sources and ATP to support growth recovery under stress conditions [40]. Km-ALE-X20 potentially regulated *TRE2* to supply resources for growth restoration and metabolic rebuilding.

To further reveal the biological functions and/or processes affected by these mutations, we performed GO and KEGG enrichment analyses. Figure 3c shows the various GO terms of mutated genes in molecular function (MF), cellular component (CC), and biological process (BP) categories (*p* < 0.05). In the BP category, mutated genes were primarily involved in carbohydrate metabolism and biosynthesis, lipid metabolism and biosynthesis, energy metabolism, nucleic acid metabolism, amino acid metabolism, and the biosynthesis of aromatic compounds. For MF, mutated genes mainly participated in DNA binding, enzymatic activity, transcriptional regulation, ADP binding, and cofactor binding. Mutated genes associated with CC were mainly localized in the extracellular region, incipient cellular bud sites, enzyme complexes, membrane protein complexes, the cell membrane, and vesicles. GO enrichment analysis indicated that mutated genes were significantly enriched in transferase activity (transferring phosphorus-containing groups), DNA binding, and the aromatic compound biosynthetic process. In the evolved strain, transferases often need to adjust their activity to meet new metabolic demands or to compensate for deficiencies in metabolic pathways. Previous studies have shown that soil salinization imposed stress on apple production, and the apple rootstock *M. halliana* responded by upregulating enzymes related to carbohydrate metabolism to supply sufficient energy and substrate [41]. Similarly, mutations in transferase-related genes in Km-ALE-X20 could have supported metabolic adaptation under lactic acid stress. In addition, mutations can affect the structure and function of DNA. DNA-binding proteins, such as transcription factors and other regulatory proteins, can recognize these changes and respond by regulating the expression of mutated genes to maintain genome stability [42]. The KEGG results (Figure 3d) shows that mutated genes were significantly enriched in the biosynthesis of secondary metabolites, carbon metabolism, and oxidative phosphorylation pathways. In *K. marxianus*, carbon metabolism plays a central role in supplying energy and precursor compounds by breaking down or converting carbon sources through pathways, including glycolysis, the tricarboxylic acid (TCA) cycle, and pyruvate metabolism [43]. The primary function of oxidative phosphorylation is to convert energy from the electron transport chain into ATP, thereby providing the chemical energy required for yeast growth and development [44]. Under lactic acid stress, lactic acid affected part of the enzyme activities of the energy and metabolite precursor supply system in Km-P, thereby inhibiting the growth and metabolite production of the strain. Km-ALE-X20 may have alleviated the damage of lactic acid by enhancing carbon metabolism and energy generation. Secondary metabolites typically include high-value compounds such as fatty acids and their esters, higher alcohols, and terpenoids, which are key contributors to aroma profiles [45]. GO and KEGG enrichment analyses results suggested that Km-ALE-X20 may have enhanced its tolerance to lactic acid stress and improved aroma production efficiency by regulating key metabolic pathways and gene expression, thereby maintaining the stability of genomes and supporting normal cell growth.

Overall, the comparative genomic analysis provided molecular-level support for the enhanced lactic acid tolerance and aroma production ability of Km-ALE-X20. Mutations in genes related to oxidative stress response (*CTA1*), membrane integrity (*ERG2*), and stress protection (*TSL1*) were critical to this phenotypic improvement. While mutations in aroma-related genes (*ARO8*, *ARO9*, *FKS2*) were also identified, their functional impact on the sensory profile will be integrated with flavoromics data in the sections below.

### 3.3. Flavoromics Analysis of K. marxianus-Fermented Milk Beer

#### 3.3.1. Dynamic Changes in Volatile Organic Compounds

**Figure 4:**
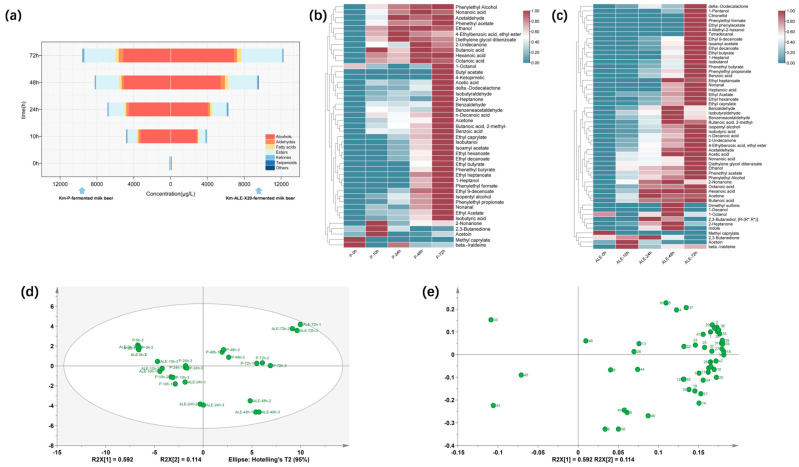
Comparison of volatile organic compounds (VOCs) during fermentation between Km-P and Km-ALE-X20-fermented milk beer. (**a**): Classification and total concentration of VOCs in Km-P and Km-ALE-X20-fermented milk beer (**b**,**c**): Heatmaps of VOCs in Km-P and Km-ALE-X20-fermented milk beer (**d**): PCA score plot of VOCs (**e**): PCA loading plot of VOCs. The numbers in the loading plot represent the names of the VOCs, as shown in Table S1.

After obtaining and validating the enhanced lactic acid tolerance and aroma production phenotypes of evolved *K. marxianus* in CDMs, we further examined the fermentation behaviors between the evolved and parental strains in the milk beer system. A total of 53 VOCs were identified during the fermentation process of milk beer. Km-P and Km-ALE-X20-fermented milk beer contained 44 and 52 VOCs, respectively (Table S2). These VOCs were categorized into seven types: alcohols, aldehydes, acids, esters, ketones, terpenes, and others. The total concentration of VOCs in Km-P and Km-ALE-X20-fermented milk beer increased over time (Figure 4a). At 0 h, the *K. marxianus* activity had not commenced, and therefore the total concentration of VOCs was relatively low compared with later fermentation stages (10 h, 24 h, 48 h, 72 h). The VOCs were dominated by acids and ketones. At subsequent fermentation stages, alcohols became the predominant VOCs, followed by esters and acids. The concentration of esters increased rapidly at 48 h and 72 h, possibly due to environmental factors such as declining pH and fluctuations in protein content in the medium [46]. During the early and middle stages of milk beer fermentation (10 h and 24 h), the total VOCs concentration of Km-P-fermented milk beer was higher than that of Km-ALE-X20-fermented milk beer. However, as fermentation progressed, lactic acid stress likely suppressed the metabolic activity of Km-P, resulting in a final VOC concentration of 9539.99 μg/L at the end of fermentation (72 h). In contrast, Km-ALE-X20 showed enhanced metabolic activity during the late fermentation phase, reaching a final VOC concentration of 12,228.81 μg/L, which was 28.18% higher than that in Km-P-fermented milk beer. The results are shown in Figure 4a–c. Moreover, the average alcohols concentration in Km-ALE-X20-fermented milk beer (6899.19 μg/L) was 33.87% higher than that in Km-P-fermented milk beer (5153.75 μg/L); the average esters concentration in Km-ALE-X20-fermented milk beer (4444.49 μg/L) was 32.43% higher than that in Km-P-fermented milk beer (3356.12 μg/L). It is worth noting that several volatile aroma compounds, including tetradecanal (citrus, musk, waxy), citronellol (floral, waxy), ethyl phenylacetate (rose), 1-pentanol (balsam), 1-decanol (floral), and indole (floral) were exclusively identified in Km-ALE-X20-fermented milk beer and were not detected in Km-P-fermented milk beer.

Principal component analysis (PCA) indicated that two principal components (PC1 and PC2) were extracted, accounting for 59.2% and 11.4%, respectively, with a cumulative contribution of 70.6% (R^2^X = 0.706). In the PCA score plot (Figure 4d), the milk beer samples at different fermentation times showed clear separation along the principal components, indicating that the VOCs in milk beer varied with fermentation time. Additionally, Km-P and Km-ALE-X20-fermented milk beer showed distinct grouping patterns, particularly at the later stages (48 and 72 h). The PCA loading plot (Figure 4e) revealed that 2,3-butanedione, ethyl butyrate, and acetoin were positioned along the negative axis of PC1, whereas phenylethyl alcohol, isoamyl alcohol, and various esters were located along the positive axis. This spatial distribution indicated that these compounds contributed to the separation of samples on PC1. Regarding PC2, compounds such as indole, (2R,3R)-2,3-butanediol, 2-nonanone, 1-decanol, dimethyl sulfone, 1-octanol, and 2-heptanone exhibited high negative loadings, while *β*-ionone, nonanal, and butyl acetate showed high positive loadings. These findings indicated that Km-P and Km-ALE-X20-fermented milk beer showed significant differences in aromatic characteristics at 48 h and 72 h, reflecting potential metabolic differences between Km-P and Km-ALE-X20 under lactic acid stress.

#### 3.3.2. OAV Analysis

The contribution of VOCs to the overall aroma of milk beer depended on both their concentrations and odor thresholds [47], which were indicated by the odor activity values (OAVs). VOCs with OAVs ≥ 1 are considered odor-active compounds, which are regarded as contributors to the aroma of the samples [48]. To further characterize the odor-active compounds in *K. marxianus*-fermented milk beer, OAVs were calculated for all detected VOCs. In this study, 25 odor-active compounds (with OAVs ≥ 1) were identified, including 3 alcohols, 4 aldehydes, 3 acids, 9 esters, and 6 ketones (Table 2). Subsequently, to examine which of these compounds differed between Km-P and Km-ALE-X20-fermented milk beer, pairwise comparisons were performed at 72 h using a two-way ANOVA model. 17 compounds were identified that exhibited significant differences (*p* < 0.05) between the two strains, which are marked with an asterisk (*) in Table 2. For clarity in the table, Km-P-fermented milk beer at 72 h was denoted as P-72h, and Km-ALE-X20-fermented milk beer at 72 h as ALE-72h. Analogous abbreviations (P-10h and ALE-10h) are used for the 10 h samples. The full results of the significance analysis are provided in Figure S2. At 72 h the OAVs of phenylethyl alcohol, isopentyl alcohol, benzeneacetaldehyde, ethyl acetate, ethyl butyrate, isoamyl acetate, ethyl hexanoate, phenethyl acetate, and 2,3-butanedione were higher than those of other odor-active compounds, indicating that these compounds played major roles in shaping the overall aroma of the milk beer. Accordingly, the sensory profile at this stage is primarily characterized by rose, fruity, creamy, and green aroma attributes. In addition, the total OAV of the Km-ALE-X20-fermented milk beer was higher than that of the Km-P-fermented milk beer, further demonstrating the enhanced aroma production capacity of the Km-ALE-X20.

Higher alcohols are important volatile aroma compounds in fermented beverages [49]. At appropriate concentrations, phenylethyl alcohol, isoamyl alcohol and pentanol can contribute to floral and fruity notes, enhancing the complexity of the aroma and sensory experience [50]. Correspondingly, phenylethyl alcohol, characterized by a rose aroma [51], exhibited the highest OAV (108,632) in the Km-ALE-X20-fermented milk beer at 72 h. The concentration of phenylethyl alcohol in Km-ALE-X20-fermented milk beer (1629.49 ± 104.92 μg/L) was 25.93% higher than that in Km-P-fermented milk beer (1293.79 ± 97 μg/L). This enhanced production of phenylethyl alcohol can be attributed to the upregulation of key enzyme genes in the Ehrlich pathway, such as *ARO8*, *ARO9*, which has been discussed in Section 3.2, Table 1. These genes encode aminotransferases that catalyze the first step in the conversion of phenylalanine to phenylethyl alcohol, leading to increased precursor availability for phenylethyl alcohol synthesis. The upregulation of *ARO8* and *ARO9* reflected varied metabolic activities in Km-ALE-X20 under lactic acid stress, reinforcing the Ehrlich pathway and thereby enhancing the aroma-producing ability.

On the other hand, esters are known to impart rich fruity, sweet, and pleasant aromas to fermented beverages [52]. Two major classes of esters were identified, including fatty acid ethyl esters and higher alcohol acetates. Ethyl acetate (OAV = 270) and ethyl butyrate (OAV = 10,020) exhibited relatively high OAVs compared to other ethyl esters in the Km-ALE-X20-fermented milk beer at 72 h. At this time, the concentrations of ethyl acetate in the Km-ALE-X20-fermented milk beer (1351.23 ± 35.66 μg/L) were 31.41% higher than that in the Km-P-fermented milk beer. The concentration of ethyl butyrate (10.02 ± 0.07 μg/L) was 69.26% higher than that in the Km-P-fermented milk beer. Among the acetate esters, isoamyl acetate (OAV = 366) and phenylethyl acetate (OAV = 73) exhibited high OAVs in the Km-ALE-X20-fermented milk beer at 72 h. The corresponding concentration of isoamyl acetate in the Km-ALE-X20-fermented milk beer (54.98 ± 1.76 μg/L) was 71.43% higher than that in the Km-P-fermented milk beer. The concentration of phenylethyl acetate (1390.10 ± 64.05 μg/L) was higher than that in the Km-P-fermented milk beer. The increased contents of ethyl esters and acetates can be attributed to the upregulation of genes involved in the biosynthetic pathways of ester precursors in the evolved strain. The upregulation of *FAS2* can promote the synthesis of fatty acids, while the upregulation of *ADH1* and *PDC2* can enhance the production of alcohols and aldehydes. In addition, the upregulation of *PYK1* and *PGK1* facilitates the synthesis of pyruvate. Similarly, *FAS2*, *ADH1*, *PDC2*, *PYK1*, and *PGK1* were also identified as potential candidate genes (Section 3.2, Table 1) contributing to the differences in aroma production between the two strains. Collectively, these results demonstrate that the evolved strain enhanced ester production through upregulation of genes involved in fatty acid, alcohol, and pyruvate metabolism, leading to increased precursor availability and a more complex and pleasant aroma profile in the milk beer.

#### 3.3.3. PLS-DA Analysis

**Figure 5:**
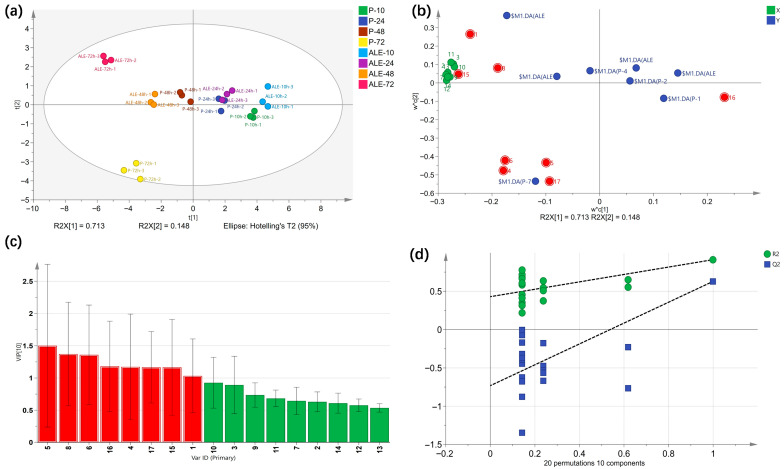
Partial least squares discriminant analysis (PLS-DA) based on 17 odor-active compounds in milk beer. (**a**): PLS-DA score plot. The legend on the right representing different times and strains. P and ALE refer to the Km-P and Km-ALE-X20-fermented-milk beer, respectively. 10, 24, 48, and 72 indicate fermentation times of 10, 24, 48, and 72 h. (**b**): PLS-DA loading plot. The numbers correspond to volatile compounds as shown in Table S3. The blue circles represent different groups of milk beer, the green circles represent selected volatile compounds and the red circles represent key volatile compounds with VIP ≥ 1. (**c**): The results of VIP. (**d**): Permutation test. The numbers in the loading plot represent the names of the VOCs, as shown in Table S3.

Partial least squares discriminant analysis (PLS-DA) was employed using 17 odor-active compounds (with OAV ≥ 1, *p* < 0.05) to identify key flavor compounds responsible for the flavor discrepancy between Km-P and Km-ALE-X20-fermented milk beer. As shown in the score plot (Figure 5a), PC1 and PC2 accounted for 71.3% and 14.8% of the total variance, respectively, with R^2^Y = 0.92 and Q^2^ = 0.696. The permutation test (Figure 5d) showed that all the blue Q^2^ values on the left side were lower than the original point on the right, and the intercept of Q^2^ on the *Y*-axis was less than 0.5. When the horizontal coordinate was 1, Q^2^ was less than R^2^, while R^2^ was very close to Q^2^. These results confirmed that the model was robust and reliable, without overfitting. On PC1, the groups of P-10, P-24, ALE-10, and ALE-24 were located in the positive direction, while P-48 and ALE-48 were located in the negative direction. On PC2, ALE-72 was located in the positive direction, and P-72 was located in the negative direction. Such distribution demonstrated that strain type significantly differentiated the aroma profile of the milk beer, consistent with previous PCA results. Variable importance for projection (VIP) score was used to further screen the key contributors to the flavor differences between Km-P and Km-ALE-X20-fermented milk beer. VIP ≥ 1 was used as the screening criterion [53]. 8 key flavor compounds were screened, namely nonanal, hexanoic acid, phenylacetaldehyde, 2,3-butanedione, isobutyraldehyde, 2-heptanone, phenethyl acetate, and ethanol (Figure 5c). Among them, nonanal, phenylacetaldehyde, isobutyraldehyde, and 2-heptanone, which are primarily aldehydes and generally impart green aroma to dairy products [54], were identified as key flavor compounds of the Km-P*-fermented milk beer, as they clustered near the 72 h Km-P-fermented milk beer in the PLS-DA loading plot (Figure 5b). Similarly, ethanol, hexanoic acid, and phenethyl acetate clustered near the 72 h Km-ALE-X20-fermented milk beer, indicating that these three compounds serve as key flavor compounds of Km-ALE-X20*-fermented milk beer.

### 3.4. Sensory Evaluation of K. marxianus-Fermented Milk Beer

**Figure 6:**
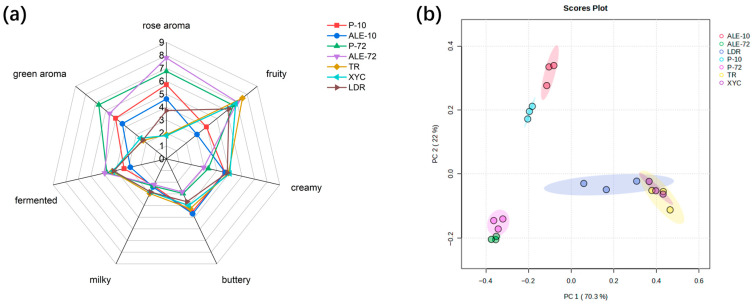
Sensory evaluation results of milk beer. (**a**) Radar chart (**b**) PCA score plot. P and ALE represent the Km-P and Km-ALE-X20-fermented milk beer, respectively, while 10 and 72 indicate 10 h and 72 h of the fermentation process, respectively. TR (Tian run milk beer), XYC (Xi yu chun milk beer), and LDR (Lan dai rose milk beer) are three rose aroma commercial milk beer used as references.

In the present study, ALE resulted in genomic mutations in *K. marxianus*. Aroma-related genes, including *ARO8*, *ARO9*, *FAS2*, and *ADH1*, were affected. These genetic changes enhanced the biosynthetic pathway of phenylethyl alcohol and regulated ester biosynthesis as well as the metabolic flux of precursor compounds. Flavoromics analysis further confirmed that, compared with Km-P-fermented milk beer, Km-ALE-X20-fermented milk beer exhibited a significant increase in both the diversity and abundance of volatile organic compounds. Therefore, to examine how the variation in volatile compounds influenced sensory characteristics, quantitative descriptive analysis (QDA) was conducted between Km-P-fermented milk beer, Km-ALE-X20-fermented milk beer, and three commercially available milk beers. The PCA score plot showed apparent distinction among the milk beer samples (Figure 6b). The three commercial milk beers clustered closely together, indicating similar sensory characteristics. This similarity aligns with the ingredient information on their labels, which shows that all three products contain comparable components such as concentrated pineapple juice and added flavoring agents. These ingredients can lead to relatively uniform aroma characteristics and make it difficult for the sensory panel to differentiate among them. Previous studies have also demonstrated that the use flavoring agents can lead to homogenized aroma profiles across different products [55]. In contrast, the Km-P and Km-ALE-X20-fermented milk beer formed distinct groups. In the radar chart of the intensity scores for seven aroma attributes (Figure 6a), the commercial milk beers obtained higher sensory scores in fruity, creamy and buttery attributes. For the Km-P and Km-ALE-X20 fermented milk beers at 10 h, the former exhibited slightly higher scores across all aroma attributes, indicating that Km-P produced a greater number of aroma compounds at this time. At 72 h, the Km-ALE-X20-fermented milk beer scored higher than Km-P-fermented milk beer in the rose aroma attribute. which is consistent with its higher concentrations of signature rose compounds such as phenylethyl alcohol and phenylethyl acetate in Km-ALE-X20-fermented milk beer. Similarly, the Km-ALE-X20-fermented milk beer also obtained higher average scores for the fruity attribute, likely due to its higher concentrations of esters. Notably, Km-P-fermented milk beer displayed a stronger green aroma intensity. This sensory outcome can be directly attributed to the higher concentrations of aldehydes, particularly nonanal and phenylacetaldehyde, which were identified as key flavor compounds for Km-P-fermented milk beer by the PLS-DA model (VIP ≥ 1). Aldehydes are well-recognized contributors to green sensory notes in fermented beverages. In summary, sensory evaluation results showed that Km-ALE-X20 significantly enhanced rose and fruity aroma attributes while it rendered a lower intensity of green aroma compared to Km-P. On the other hand, the Km-P-fermented milk beer presented a more balanced and mellow aroma profile. These results indicate that obtaining aroma-enhancing strains does not necessarily lead to improved overall flavor balance. Specifically, the ALE-based strain selection approach has certain limitations. ALE is usually performed under optimal growth conditions for the strain, such as optimal substrates, temperature, and dissolved oxygen levels. Under these conditions, ALE effectively directs metabolic flux toward favorable metabolic pathways, such as the phenylethyl alcohol biosynthesis pathway and ester biosynthesis pathways identified in this study, but it may simultaneously reduce metabolic flexibility, resulting in a decreased abundance of other aroma compounds. When transitioning from laboratory-scale experiments to actual industrial production, changes in cultivation conditions or prolonged storage periods may cause the evolved strains to exhibit a limited capacity for metabolic flux redistribution, leading to the accumulation of metabolic intermediates or the suppression of certain odor-active compounds, thereby increasing the risk of off-flavor formation. To make the full use of such flavor-enhanced strains, co-fermentation with other microbial strains or optimization of the fermentation process is warranted.

## 4. Conclusions

In this study, a lactic acid-tolerant and flavor-enhanced *K. marxianus* evolved strain, Km-ALE-X20, was obtained via adaptive laboratory evolution. Compared with the parental strain, Km-ALE-X20 exhibited a 16-fold increase in OD_600_ and a 28-fold increase in phenylethyl alcohol production under 20 g/L lactic acid stress in a chemically defined medium. Comparative genomics analysis revealed that the improved acid stress resistance and aroma-producing phenotypes were associated with mutations in several key genes. Specifically, *CTA1*, *TSL1*, and *ERG2* contributed to oxidative stress resistance, trehalose metabolism, and membrane stabilization, while *ARO8*, *ARO9*, and *FKS2* were involved in the synthesis of higher alcohols and fatty acids. Furthermore, when applying Km-ALE-X20 to ferment milk beers, it improved both the diversity and contents of aroma compounds compared with Km-P-fermented milk beer. Specifically, Km-ALE-X20-fermented milk beer showed 33.87% and 32.43% higher concentrations of alcohols and esters, respectively. Furthermore, sensory evaluation showed that Km-ALE-X20-fermented milk beer exhibited enhanced rose and fruity aroma attributes, whereas the Km-P-fermented milk beer possessed a more balanced aroma profile. From an industrial perspective, the rose aroma-enhanced *K. marxianus* strain obtained through ALE shows commercial potential for improving the flavor quality and product differentiation of fermented dairy products. In addition, this approach can be applied to aroma-producing food microorganisms used in food fermentation beyond dairy products, thereby providing a versatile platform for metabolic optimization of food-related strains. More importantly, as ALE does not involve genetic modification, the resulting strains are compatible with current food safety regulations. In summary, this work represents the first application of adaptive laboratory evolution to enhance the characteristic rose-like aroma of *K. marxianus* and presents an efficient workflow integrating ALE, flavoromics, and sensory evaluation for the development of flavor-enhanced *K. marxianus* strains for milk beer. This workflow also exhibits broad applicability and can be extended to other aroma-producing food microorganisms. In future studies, the ALE strategy may be applied to a wider range of aroma-producing food microorganisms and fermentation substrates to evaluate its robustness. Furthermore, under the more complex conditions encountered in industrial-scale fermentation, additional process-level optimization may be required, including adjustments to fermentation parameters such as temperature, fermentation time, and inoculation level, together with co-fermentation strategies involving other aroma-producing food microorganisms, to construct a more harmonious overall flavor profile in *K. marxianus*-fermented milk beer.

## Figures and Tables

**Table 1:** Key mutated genes of Km-ALE-X20 compared with Km-P.

Gene	Function	Mutation Type	Protein-Altering	Non-Protein-Altering
Mutated genes associated with acid resistance				
*CTA1*	Catalasa	SNP, indel	2	17
*YBP1*	Oxidative-stress sensor that mediates the activation of the *Cap1* transcription factor	SNP, indel	7	13
*POS5*	NADH kinase	SNP	1	0
*IPT1*	Inositol Phosphoryl Transferase	SNP	3	9
*ERG2*	C-8 sterol isomerase	SNP, indel	1	27
*ERG7*	Lanosterol synthase	SNP	1	2
*VMA16*	V-ATPase V_0_ subunit c	SNP, indel	3	8
*PDR12*	ATP-binding cassette (ABC) transporter	SNP	3	4
*TSL1*	Trehalose-6-phosphate synthase regulatory subunit	SNP, indel	6	16
*TRE2*	Neutral trehalase	SNP, indel	4	11
Mutated genes associated with aroma production				
*ARO8*	Aromatic aminotransferase I	SNP, indel	0	5
*ARO9*	Aromatic aminotransferase II	SNP, indel	0	3
*ADH1*	Alcohol dehydrogenase 1	indel	0	1
*FAS2*	Fatty acid synthetase subunits *α*	indel	0	1
*PYK1*	Pyruvate kinase 1	SNP, indel	0	13
*PDC2*	Pyruvate decarboxylase 2	SNP	2	1
*ILV1*	Threonine deaminase	SNP, indel	2	11
*ILV2*	Acetolactate synthase	indel	0	4
*BAT1*	Mitochondrial branched-chain amino acid aminotransferase	SNP	0	1
*LEU3*	Leucine-responsive regulatory protein	SNP, indel	5	10

**Table 2:** Odor-active compounds identified in *K. marxianus*-fermented milk beer (* *p* < 0.05).

Compounds	Odor Descriptions	Thresholds (μg/L)	OAV			
			P-10 h	P-72 h	ALE-10 h	ALE-72 h
Ethanol *	alcoholic, ethereal	3500	<1	<1	<1	1
Isopentyl alcohol *	fruity, banana, malt	4	30	195	20	255
Phenylethyl alcohol *	rose	0.015	50,654	86,252	37,269	108,632
Acetaldehyde	pungent, ethereal, fruity	0.7	94	205	65	257
Isobutyraldehyde *	fresh, floral, green, malt	0.32	18	108	17	33
Nonanal *	rose, orange, green	0.32	6	25	<1	1
Benzeneacetaldehyde *	rose, honey, green	0.3	487	991	163	451
Isobutyric acid *	sharp, pungent, sour	10	1	8	<1	11
Butanoic acid, 2-methyl-	pungent, cheese	10	1	3	<1	4
Hexanoic acid *	sour, cheese	35.6	1	1	<1	2
Ethyl acetate *	apple, banana, fruity	5	46	205	39	270
Ethyl butyrate *	fruity pineapple	0.001	<1	5920	<1	10,020
Isoamyl acetate *	sweet fruity banana	0.15	<1	213	<1	366
Ethyl hexanoate *	fruity pineapple banana	0.01	<1	2699	<1	3434
Ethyl heptanoate	fruity pineapple	1.9	<1	1	<1	1
Ethyl caprylate *	pineapple, banana, pear	5	<1	30	<1	41
Ethyl decanoate *	fruity apple grape	5	<1	8	<1	11
Phenethyl acetate *	rose, fruity, tropical	19	33	62	25	73
δ.-Dodecalactone	peach, coconut	0.46	14	79	13	78
Acetone	ethereal, apple, pear	40	<1	2	<1	1
2,3-Butanedione *	butter, sweet, creamy	0.05	642	261	461	151
2-Heptanone *	fruity, green, fatty	1	11	21	9	9
Acetoin	sweet, buttery creamy	14	2	1	3	<1
2-Nonanone	fresh, sweet, green, herbal	5	2	2	2	2
2-Undecanone	Fruity, creamy, fatty, floral	5.5	<1	1	<1	1

## Data Availability

The original contributions presented in this study are included in the article/Supplementary Materials. Further inquiries can be directed to the corresponding authors.

## Supplementary Materials

The following supporting information can be downloaded at https://www.mdpi.com/article/10.3390/foods15020229/s1. Figure S1: Standard curve of phenylethyl alcohol; Figure S2: Significance analysis of 17 odor-active compounds; Table S1: The list of the different VOCs used in the PCA model; Table S2: The VOCs in Km-P and Km-ALE-X20-fermented milk beer; Table S3: List of the 17 odor-active compounds used in the PLS-DA model.
