# Supplementary material for: Enhancing the Signature Rose Aroma of Kluyveromyces marxianus-Fermented Milk Beer via Adaptive Laboratory Evolution
Source: Foods. 2026 Jan 8;15(2):229. doi: 10.3390/foods15020229 (PMC12840503; doi:10.3390/foods15020229)
Supplement: Supplementary file 1 [file foods-15-00229-s001.zip › foods-4057718-supplementary.pdf]

Supplementary Materials

Table S1. A list of the different VOCs used in the PCA model.

NO.	Compounds	NO.	Compounds	NO.	Compounds	NO.	Compounds	NO.	Compounds
1	Ethanol	11	Acetaldehyde	21	Hexanoic acid	31	Ethyl hexanoate	41	δ-Dodecalactone
2	Isobutyl alcohol	12	Isobutyraldehyde	22	Heptanoic acid	32	Ethyl heptanoate	42	Acetone
3	Isopentyl alcohol	13	Nonanal	23	Benzoic acid	33	Methyl caprylate	43	2,3-Butanedione
4	1-Pentanol	14	Benzaldehyde	24	Octanoic acid	34	Ethyl caprylate	44	2-Heptanone
5	4-Methyl-2-hexanol	15	Benzeneacetaldehyde	25	Nonanoic acid	35	Ethyl decanoate	45	Acetoin
6	2,3-Butanediol, [R-(R*,R*)]	16	Tetradecanal	26	n-Decanoic acid	36	Ethyl 9-decenoate	46	2-Nonanone
7	1-Heptanol	17	Acetic acid	27	Ethyl Acetate	37	Phenylethyl formate	47	2-Undecanone
8	1-Octanol	18	Isobutyric acid	28	Butyl acetate	38	Phenethyl acetate	48	*β*.-Iraldeine
9	1-Decanol	19	Butanoic acid	29	Ethyl butyrate	39	Phenylethyl propionate	49	Dimethyl sulfone
10	Phenylethyl Alcohol	20	Butanoic acid, 2-methyl-	30	Isoamyl acetate	40	Phenethyl butyrate	50	Indole

Figure S1. Standard curve of phenylethyl alcohol. The concentration of phenylethyl alcohol was determined using a solvent extraction method. Briefly, 1.5 mL of the fermentation broth was centrifuged to remove cells, and 1 mL of the supernatant was collected. Chloroform was added to the supernatant, and the mixture was thoroughly vortexed. Phase separation was achieved by centrifugation at 3,000 rpm, and the lower chloroform phase was collected for absorbance measurement at 254 nm.

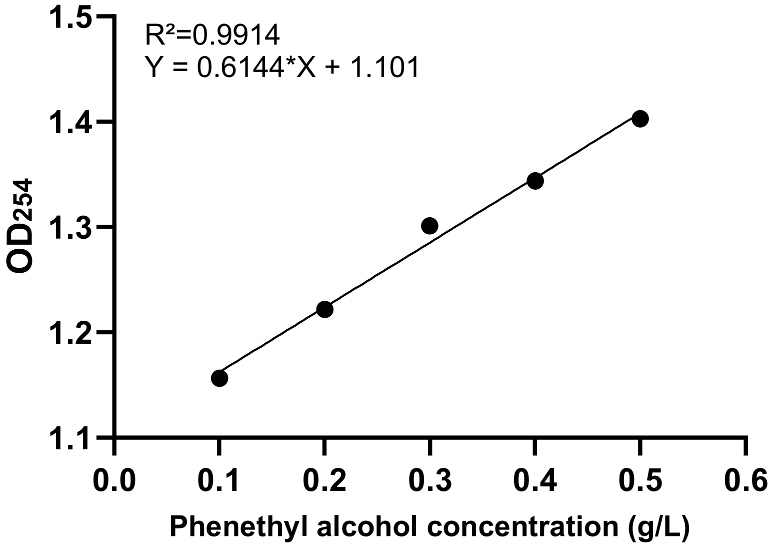

Table S2. The VOCs in Km-P and Km-ALE-X20-fermented milk beer.

Category	Compounds	RT	RI	CAS	P Group Concentration (μg/L)					ALE Group Concentration (μg/L)				
					p-0h	p-10h	p-24h	p-48h	p-72h	ALE-0h	ALE-10h	ALE-24h	ALE-48h	ALE-72h
	Ethanol	8.775	940	64-17-5	ND	2,403.2±157.96	3,105.58±271.15	3,175.9±167.55	2,939.51±160.52	ND	2,216.71±64.65	2,713.6±123.3	3,214.65±69.57	4,036.7±94.41
	Isobutanol	14.938	1102	78-83-1	ND	ND	21.12±2.76	81.63±16.71	133±22.99	ND	ND	25.44±4.05	83.91±21.63	198.45±9.69
	Isopentyl alcohol	20.236	1213	123-51-3	0.49±0.07	120.48±8.26	318.07±36.86	600.78±127.95	782.71±44.56	0.49±0.02	82.22±17.86	364.68±62.75	811.91±33.16	1,023.17±47.13
	1-Pentanol	22.24	1255	71-41-0	ND	ND	ND	1.62±0.36	ND	ND	ND	ND	0.51±0.27	2.38±0.31
	4-Methyl-2-hexanol	25.462	1323	2313-61-3	ND	ND	ND	ND	ND	ND	ND	ND	ND	4.76±0.23
Alcohols	2,3-Butanediol, [R-(R*,R*)]	27.479	1367	24347-58-8	ND	ND	ND	ND	ND	ND	ND	0.77±0.16	0.6±0.02	ND
	1-Heptanol	31.604	1459	111-70-6	ND	ND	ND	2.39±0.49	3.45±0.34	ND	ND	ND	1.94±0.39	3.29±0.72
	1-Octanol	35.962	1562	111-87-5	1.07±0.05	0.78±0.01	0.62±0.1	0.77±0.17	1.3±0.05	1.09±0.05	0.83±0.26	1.02±0.42	1.16±0.2	0.95±0.35
	1-Decanol	40.099	1664	112-30-1	ND	ND	ND	ND	ND	ND	ND	ND	3.02±0.23	ND
	Phenylethyl Alcohol	49.491	1916	60-12-8	3.7±0.89	759.82±33.2	1,098.33±41.98	1,234.15±13.27	1,293.79±97	2.28±0.42	559.04±24.24	970.93±28.62	1,354.96±21.76	1,629.49±104.92
	Acetaldehyde	4.714	676	75-07-0	9.48±0.88	65.8±15.61	144.58±19.61	131.16±3.58	143.75±6.38	8.01±0.76	45.83±6.23	98.94±17.49	165.61±21.8	180.54±39.07
	Isobutyraldehyde	5.775	799	78-84-2	ND	5.77±1.26	10.75±1.51	9.52±1.06	34.61±2.04	ND	5.5±0.63	9.57±0.78	18.4±1.4	10.64±0.67
	Nonanal	28.94	1399	124-19-6	ND	1.99±0.72	0.76±1.32	6.07±2.28	8±0.76	ND	ND	ND	0.37±0.07	0.52±0.09
Aldehydes	Benzaldehyde	35.025	1539	100-52-7	ND	5.93±1.09	5.25±1.03	6.21±1.68	14.57±1.26	ND	2±0.32	9.62±2.73	16.53±2.29	9.37±0.45
	Benzeneacetaldehyde	39.746	1655	122-78-1	ND	146.11±12.6	128.31±23.04	92.07±16.73	297.5±31.09	ND	48.96±7.55	87.43±11.53	227.21±23.02	135.31±7.37
	Tetradecanal	42.205	1718	124-25-4	ND	ND	ND	ND	ND	ND	ND	ND	ND	1.17±0.09
	Acetic acid	31.762	1463	64-19-7	2.7±0.83	17.46±2.04	11.7±0.14	20.24±11.64	48.7±9.54	3.1±1.51	18.83±0.68	20.39±5.11	40.49±7.29	43.76±2.15
	Isobutyric acid	36.406	1572	79-31-2	ND	11.16±2.7	22.51±2.82	53.12±7.73	89.44±9.66	ND	6.64±0.5	33.8±6.16	85.57±5.41	115.39±9.04
	Butanoic acid	38.954	1635	107-92-6	1.06±0.13	7.14±0.59	4.97±0.74	7.12±1.24	6.64±1.77	1.55±0.42	5.04±0.78	9.98±2.02	10.53±0.77	11.37±1.06
	Butanoic acid, 2-methyl-	40.52	1674	116-53-0	ND	11.9±0.96	16.54±1.33	21.86±4.06	33.33±10.6	ND	2.62±0.75	18.75±4.76	46.84±2.45	40.84±2.54
Acids	Hexanoic acid	47.078	1849	142-62-1	6.4±0.95	51.24±3.69	44.48±1.64	60.7±5.73	61.89±6.2	11.86±3.65	27.71±5.1	72.58±14.98	73.6±5.85	72.33±4.14
	Heptanoic acid	50.85	1956	111-14-8	ND	ND	ND	ND	ND	ND	ND	ND	2.62±0.44	3.64±0.25
	Benzoic acid	53.183	2024	65-85-0	ND	20.24±10.32	18.15±0.87	29.41±13.1	45.24±39.04	ND	ND	14.38±11.57	29.79±11.47	54.04±11.04
	Octanoic acid	54.392	2061	124-07-2	4.84±0.84	45.69±5.32	41.24±8.75	58.77±8.67	54.08±7.63	12.32±3.95	21.51±4.08	61.75±8.3	61.79±9.76	71.98±8.74
	Nonanoic acid	57.787	2167	112-05-0	ND	6.03±2.07	10.83±1.81	12.96±3.74	11.67±0.63	ND	7.98±4.32	8.93±1.44	11.11±1.69	19.87±1.66
	n-Decanoic acid	61.338	2282	334-48-5	0.99±0.17	7.63±1.2	6.42±3.12	8.36±2.79	12.29±2.1	2.21±0.57	3.29±1.22	8.18±3.23	11.1±2.54	15.52±4.06

Category	Compounds	RT	RI	CAS	P Group Concentration (μg/L)					ALE Group Concentration (μg/L)				
					p-0h	p-10h	p-24h	p-48h	p-72h	ALE-0h	ALE-10h	ALE-24h	ALE-48h	ALE-72h
	Ethyl Acetate	7.155	878	141-78-6	ND	233.9±16.21	273.96±28.5	645.43±94.63	1,028.24±101.5	ND	199±12.23	224.15±20.03	1,050.16±25.93	1,351.23±35.66
Esters	Butyl acetate	11.165	1013	123-86-4	ND	ND	ND	ND	8.8±1.33	ND	ND	ND	ND	ND
	Ethyl butyrate	12.022	1033	105-54-4	ND	ND	ND	3±1.83	5.92±0.25	ND	ND	ND	4.63±0.6	10.02±0.07

	Isoamyl acetate	15.614	1116	123-92-2	ND	ND	5.41±0.78	20±1.37	32.07±1.04	ND	ND	5.45±1.14	29.71±9.09	54.98±1.76
	Ethyl hexanoate	21.006	1229	123-66-0	ND	ND	4.6±0.98	14.56±2.93	26.99±0.99	ND	ND	5.2±0.84	24.29±0.95	34.34±1.95
	Ethyl heptanoate	25.954	1334	106-30-9	ND	ND	ND	1.6±0.41	1.96±0.13	ND	ND	ND	2.62±0.16	2.47±0.27
	Methyl caprylate	28.375	1387	111-11-5	5±1.69	ND	ND	ND	ND	8.37±0.28	ND	ND	ND	ND
	Ethyl caprylate	30.562	1436	106-32-1	ND	3.95±0.06	44.22±4.46	106.25±9.98	154.92±9.77	ND	1.77±0.31	38.52±4.36	147.79±2.9	206.99±8
	Ethyl decanoate	39.209	1641	110-38-3	ND	ND	7.4±0.45	23.99±1.07	42.79±4.77	ND	ND	5.07±0.39	33.84±2.27	58.33±4.81
	Ethyl 9-decenoate	41.275	1693	67233-91-4	ND	ND	1.1±0.04	3.78±0.27	4.28±0.57	ND	ND	1.05±0.08	4.02±0.15	7.81±0.65
	4-Ethylbenzoic acid, ethyl ester	44.752	1785	36207-13-3	ND	83.2±12.39	115.96±47.23	94.87±95.17	81.4±8.31	ND	20.53±1.9	71.24±25.46	99.62±29.31	139.23±27.9
	Phenylethyl formate	45.063	1793	104-62-1	ND	ND	ND	4.17±0.45	5.95±0.27	ND	ND	ND	ND	6.19±3.52
	Phenethyl acetate	46.258	1826	103-45-7	1.11±0.4	629.38±13.03	989.52±43.35	947.4±11.15	1,185.02±61.99	0.43±0.1	480.43±19.34	920.15±24.75	984.19±52.81	1,390.1±64.05
	Phenylethyl propionate	48.522	1889	122-70-3	ND	33.66±1.32	252.78±1.47	543.44±31.38	694.65±50.38	ND	16.02±0.98	250.81±25.61	619.01±33.52	1,013.87±88.05
	Phenethyl butyrate	51.322	1969	103-52-6	ND	ND	ND	17.41±3.82	30.62±3.79	ND	ND	7.83±1.27	20.78±2.45	50.42±9.77
	Ethyl phenylacetate	52.783	2012	101-97-3	ND	ND	ND	ND	ND	ND	ND	ND	ND	48.32±10.2
	Diethylene glycol dibenzoate	58.963	2204	120-55-8	ND	12.36±1.88	19.29±2.84	17.62±2.14	15.74±2.02	ND	18.15±4.79	15.85±4.6	19.11±9.5	33.98±17.86
	delta.-Dodecalactone	60.317	2249	713-95-1	1.82±0.08	6.86±0.59	7.24±1.09	16.49±9.39	36.77±2.29	2.54±0.19	5.99±0.71	17.14±3.73	10.49±5.07	36.21±2
Ketones	Acetone	5.967	810	67-64-1	ND	27.57±16.22	44.47±18.65	48.77±31.7	92.31±21.62	ND	22.65±5.26	57.6±28.58	52.01±17.3	53.22±13.93
	2,3-Butanedione	9.989	980	431-03-8	17.09±1.32	32.14±8.06	19.14±0.52	11.68±1.08	13.07±0.78	19.05±2.74	23.05±3.64	26.89±2.25	15.82±3.32	7.58±0.38
	2-Heptanone	18.381	1174	123-51-3	8.99±0.12	11.66±1.14	12.49±1.1	10.12±0.68	21.62±2.98	9.16±0.2	9.36±3.58	10.75±1.53	11.32±1.94	9.68±0.76
	Acetoin	24.372	1300	513-86-0	11.58±0.71	32.48±2.06	7.15±1.07	8.33±1.21	15.44±1.69	12.57±2.03	43.14±9.95	18.18±3.59	9±1.64	7.57±1.34
	2-Nonanone	28.819	1396	821-55-6	8.06±0.32	11.88±0.41	8.72±0.73	10.07±1.36	10.35±0.59	8.3±0.14	10.93±2.68	13.14±2.71	13.81±0.75	11.61±0.19
	2-Undecanone	38.124	1614	112-12-9	2.84±0.09	4.83±0.1	4.3±0.5	6.92±0.47	6.26±0.92	3.08±0.09	4.05±0.98	5.8±0.17	7.75±0.34	9.17±0.42

Category	Flavor Compounds	RT	RI	CAS	P Group Concentration (μg/L)					ALE Group Concentration (μg/L)				
					p-0h	p-10h	p-24h	p-48h	p-72h	ALE-0h	ALE-10h	ALE-24h	ALE-48h	ALE-72h
Terpenes	Citronellol	44.193	1770	106-22-9	ND	ND	ND	ND	ND	ND	ND	ND	ND	1.23±0.03
	beta.-Iraldeine	62.657	2327	127-43-5	2.54±0.96	1.08±0.43	2.35±0.14	1.41±0.68	1.51±0.91	0.4±0.02	2.84±0.56	0.97±0.45	1.79±1.03	2.12±0.16
others	Dimethyl sulfone	19.876	1206	67-71-0	ND	ND	ND	ND	ND	ND	ND	ND	7.11±3.35	ND
	4-Ketopimelic	12.51	1045	502-50-1	ND	ND	ND	ND	ND	ND	ND	ND	ND	7.88±3.77
	Indole	53.661	2039	120-72-9	ND	ND	ND	ND	ND	ND	ND	5.17±2.85	8.79±0.55	ND

Figure S2. Significance analysis of 17 odor-active compounds.

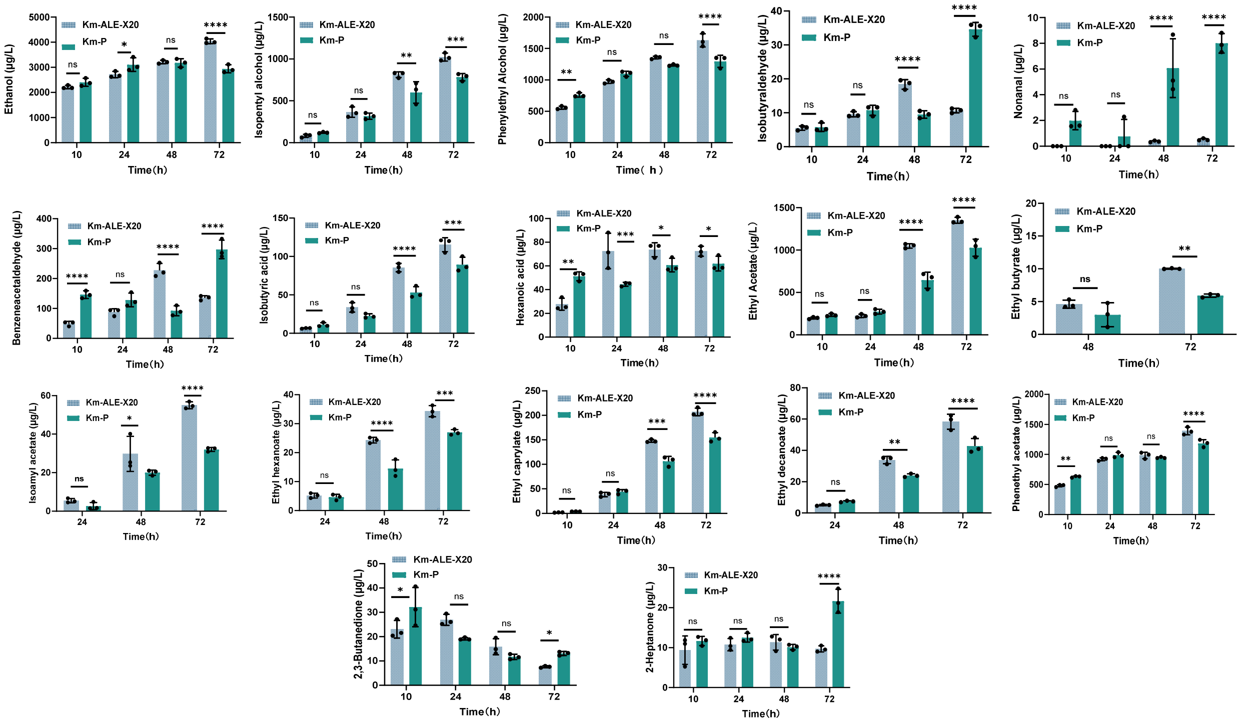

Table S3. List of the 17 odor-active compounds used in the PLS-DA model.

NO.	Compounds	NO.	Compounds	NO.	Compounds
1	Ethanol	7	Isobutyric acid	13	Ethyl caprylate
2	Isopentyl alcohol	8	Hexanoic acid	14	Ethyl decanoate
3	Phenylethyl Alcohol	9	Ethyl Acetate	15	Phenethyl acetate
4	Isobutyraldehyde	10	Ethyl butyrate	16	2,3-Butanedione
5	Nonanal	11	Isoamyl acetate	17	2-Heptanone
6	Benzeneacetaldehyde	12	Ethyl hexanoate		
